# Studies on the ethnopharmacology, antimicrobial activity, and toxicity of *Catha edulis* (Vahl.) Endl., in *Sprague Dawley* rats

**DOI:** 10.12688/f1000research.109243.2

**Published:** 2022-08-18

**Authors:** Kevin Kariuki Githua, Timothy Elias Maitho, Joseph Mwanzia Nguta, Mitchel Otieno Okumu

**Affiliations:** 1 Health, County Government of Embu, Embu, Embu, +254, Kenya; 2 Public Health, Pharmacology and Toxicology, University of Nairobi, Nairobi, Nairobi, 254, Kenya; 3 Pharmacy, Jaramogi Oginga Odinga Teaching and Referral Hospital, Kisumu, Kisumu, +254, Kenya

**Keywords:** Catha edulis, khat, miraa, antimicrobial activity, toxicity, Mbeere South

## Abstract

**Background: **The Mbeere South community of Embu County consume leaves of *Catha edulis* for its stimulant and euphoretic actions. Other indigenous uses of the plant are undocumented. Information on the pharmacology and safety of this plant is also scanty. This study aimed to document the ethnopharmacology, antimicrobial properties, and toxicity of *C. edulis* leaves collected from the Mbeere South community in Kenya.

**Methods: **Ethnopharmacological data was collected from 35 informants using semi-structured questionnaires. Leaf extracts of *C. edulis *were prepared using acetone, water, and methanol. The antimicrobial properties of these extracts were evaluated against *Bacillus cereus, Staphylococcus aureus, Escherichia coli, Pseudomonas aeruginosa, *and *Candida albicans*. The toxicity of the aqueous extract was determined using hematological, biochemical, and histopathological parameters in male and female *Sprague Dawley* rats at 250 mg/kg, 500 mg/kg, and 1000 mg/kg doses over 28 days. *p<0.05* was considered significant.

**Results: **All informants were male, most were married, >50 years old, with >10 years of experience. The sources, local names, preparation, storage conditions, indications, frequency of use, dosage, and side effects of *C. edulis* were documented. All extracts were ineffective against *E. coli*, *P. aeruginosa, *and *C. albicans. *They had limited efficacy against *B. cereus *and *S. aureus.* Significant differences were observed in the hematological and biochemical parameters of rats at the tested doses. Low, intermediate, and high doses of the aqueous extract of *C. edulis* produced local congestion of the cardiac and hepatic vessels. Localized interstitial connective tissue proliferation, multifocal kidney interstitial hemorrhage, and localized tubular epithelium necrosis were also observed in female rats.

**Conclusions: **The ethnobotanical uses of *C. edulis* among the Mbeere South community were documented for the first time. Limited antimicrobial efficacy and toxicity at high doses limit the use of leaves of *C. edulis*.

## List of abbreviations

ANOVA: Analysis of Variance

AR: Analytical Reagent

ARRIVE: Animal Research Reporting of *In vivo* experiments

BW: Body weight

CLSI: Clinical and Laboratory Standard Institute

CTMDR: Center for Traditional Medicine and Drug Research

DMSO: Dimethyl Sulphoxide

EDTA: Ethylene Diamine Tetra Acetic Acid

HGB: Haemoglobin

HPLC: High-Performance Liquid Chromatography

KEMRI: Kenya Medical Research Institute

LYM: lymphocytes

MBC: Minimum bactericidal concentration

MCHC: Mean corpuscular hemoglobin concentration

MCV: Mean corpuscular volume

MHB: Muller Hinton Broth

MIC: Minimum Inhibitory Concentration

MS: Microsoft

PHPT: Public Health, Pharmacology and Toxicology

PLT: Platelets

OECD: Organization for Economic Cooperation and Development

RBC: Red blood cells

TSA: Trypton Soy Agar

UK: United Kingdom

WBC: White blood cells

## Introduction

**Figure 1.**
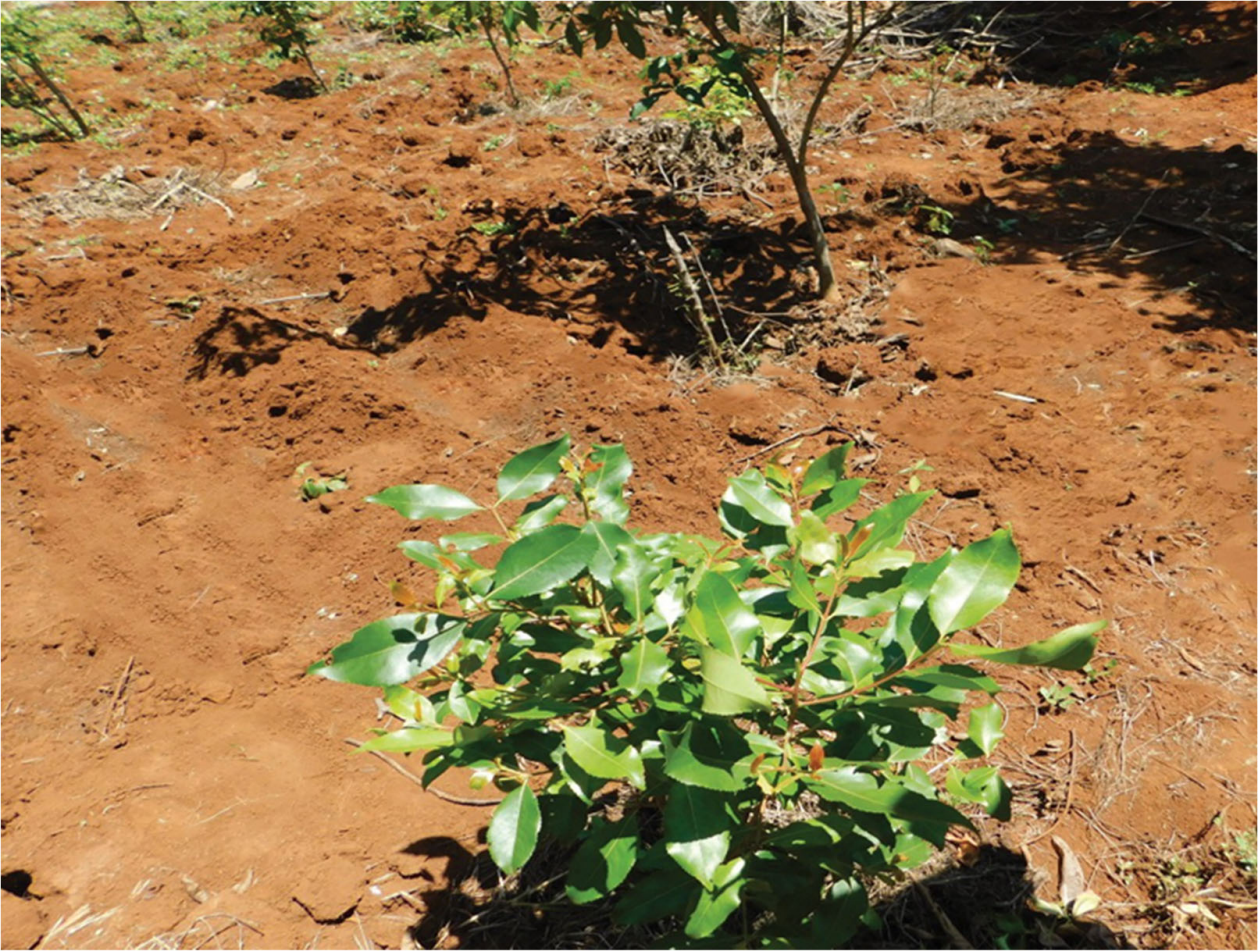
*Catha edulis* (Vahl.) Endl.

*Catha edulis* (Vahl.) Endl is a small shrub with glossy green leaves, a whitish bark, and a small crown ^1^^,^^2^ ( Figure 1).

The flowers are white and arranged in small branches. ^1^^,^^2^ The plant grows in Africa and the Southwest-Arabian peninsula. ^3^^,^^4^ The wide geographic distribution of the plant has made it go by many names, e.g. *Catha, Kat, Qat, Chat, Ciat, Tschat, Miraa, Murungu,* and Arabian or Abyssinian tea. ^5^^,^^6^ Recent estimates suggest that up to 20 million people chew the plant regularly to enjoy its psychostimulant effects. ^7^^,^^8^ The main compounds in the plant are Cathinone and Cathine. ^6^ These compounds have been associated with amphetamine-like effects; thus, *C. edulis* is scheduled in some countries and listed in the 1971 United Nations Convention on Psychotropic Substances under Schedules I and III. ^6^ The use of *Khat* in the United Kingdom (UK) is banned following its classification as a class C drug. ^9^ Anyone found to possess the stimulant will be fined 60 pounds, while suppliers risk a 14-year jail term. ^9^

Fresh vegetable material (stems, leaves, and flower buds) is chewed, and the juice of the masticated material is swallowed while the residues are spat out. An individual may consume up to 200 grams of the leaves of *Catha edulis* (about one bundle) every session. ^6^ The initial pharmacological effects include elevated blood pressure, euphoria, elation/excitability, increased alertness, and arousal. ^7^^,^^10^^,^^11^ These effects are later replaced by depression, irritability, anorexia, and insomnia. ^7^^,^^10^^,^^11^ Chewing Khat has also been associated with psychological dependence. ^12^^,^^13^ Bogale *et al*. reported that subchronic oral administration of *Khat* showed an enhanced locomotor activity, reduced social interaction, and impaired cognitive function, which demonstrated that long-term use of *Khat* may be related to schizophrenic-like symptoms. ^7^ Ethnopharmacological, toxicological, and antimicrobial data on *Catha edulis* is limited. ^3^^,^^14^^–^^17^ In Kenya, some of the ethnopharmacological uses of *Catha edulis* that have been documented include; treatment of helminthiasis, toothache, asthma, erectile dysfunction, general body pain, gonorrhoea, heartburn, influenza, pneumonia, stomach upset, coughing, diarrhoea, and fatigue. ^17^ To the best of our knowledge, this study is among a few to report on the ethnopharmacology, antimicrobial activity, and toxicity of *Catha edulis* leaves consumed by the Mbeere South community of Embu County in Kenya.

## Methods

### Ethical considerations

The National Commission for Science, Technology, and Innovation approved this research (REF: 760323) on 17 ^th^ June 2021. All participants gave written consent to participate in the study and confidentiality was upheld throughout the study. Approval for conducting the animal experiments was gained from the Faculty of Veterinary Medicine Biosafety, Animal Use and Ethics Committee, University of Nairobi (REF BAUEC/2020/256) on 8th January 2020.

Animal experiments were conducted under the Biosafety, Animal Use, and Ethics Committee guidelines of the University of Nairobi. In order to minimize harm to the animals, the Organization for Economic Cooperation and Development (OECD) 407 (2008) guidelines were strictly adhered to. The Animal Research Reporting of *In* vivo Experiments (ARRIVE) guidelines were also used. ^51^

### Ethnopharmacological field survey and collection of plant materials

Snowballing sampling technique as described by Kiunga and colleagues ^17^ was used to conduct the survey. Initial validity testing was carried out at the departmental level and preliminary pilot testing using six local herbalists was carried out in the neighboring Sub County of Mbeere North. Feedback from the six herbalists was the basis for reliability testing i.e. for internal consistency, test-retest, and inter-rater. Minor changes were implemented resulting from the preliminary testing. The inclusion criteria were local herbalists and local farmers while the exclusion criteria was local farmers with less than 4 years of experience in traditional herbal medicine. A community health volunteer was used as a go between the interviewers and the interviewees (local herbalists and farmers). Only willing participants were interviewed, and confidentiality was maintained throughout the study. Information on the traditional uses of *C. edulis* was generated strictly from the community of Mbeere South through open and closed ended questionnaires targeting the local herbalists and farmers. The questionnaire used can be found as *Extended data.* ^50^

Collection of the plant leaves was conducted by a team comprising local farmers and herbalists with a good knowledge of the area. A sample of the freshly picked leaves of *C. edulis* was arranged between newspapers, packaged in a carton, and transported to the National Museum of Kenya herbarium for voucher specimen identification, authentication, and deposition. The specimen was identified as *Catha edulis* with a reference number of NMK/BOT/CTX/1/2. The remaining leaves were packaged in woven bags and transported to the public health, pharmacology, and toxicology laboratory for drying.

### Extraction

*Acetone extract*

1214.8 grams of *Catha edulis* leaves powder was weighed using a top pan balance (Mettler PE 2000) and put in an extraction jar. 3000 ml of 99.5% (analytical reagent) AR grade acetone (Loba Chemie PVT LTD B326802002) was added to the powder in an extraction chamber and stirred using a glass rod. The preparation was left to macerate for 72 hours under constant stirring using a magnetic stirrer (SH-2). The mixture was then filtered using a Whatman filter paper No.1 into a round-bottomed flask. The filtrate was put on a rotary evaporator (Buchi Rotovapor R) for concentrated evaporation at 35°C for 2 hours. The resulting content was transferred to 2 amber glass bottles, sealed with an aluminum foil, and placed on a sand bath for further drying at 40°C.

*Methanol extract*

300 g of the *Catha edulis* powder extract was weighed using a top pan balance (Mettler) and put in an extraction jar. 1000 ml of 99.8% High-Performance Liquid Chromatography (HPLC) grade Methanol (Loba Chemie PVT LTD UN NO. 1230) was added to the extraction jar, and the mixture was stirred using a glass rod. The mixture was then put in a magnetic stirrer for 72 hours and filtered into a round-bottomed flask using Whatman filter paper No.1. The filtrate was put on a rotary evaporator (Buchi) for concentrated evaporation at 50°C, and the process took 2 hours. The resulting content was placed in an amber glass bottle and transferred to a sand bath for further drying at 40°C.

*Aqueous extract*

This process was carried out at Kenya Medical Research Institute (KEMRI) Center for Traditional Medicine and Drug Research (CTMDR) laboratory. 1180 g of *Catha edulis* powder was soaked in 6500 ml of distilled water. It was heated in a water bath for 2 hours at 70°C to activate extraction. The mixture was filtered using cotton gauze and then passed through a ball of cotton wool. 5.1 liters of the filtrate obtained were put in freeze-drying flasks and freeze-dried using carbon ice. 99.5% AR grade acetone (Loba Chemie PVT LTD B326802002) was added to the dry ice pellets to enhance the quick solidification of the extract. The solidified extract was put in a freeze dryer for 48 hours to separate the extract from the water.

### Antimicrobial assay

*Preparation of the culture media*

6.3 g of Muller Hinton Broth (MHB) (HIMEDIA REF M391-500G) was mixed with 300ml of distilled water. The solution was then warmed using a microwave for about 2 minutes to help the MHB dissolve well. 2 ml of the solution was poured into test tubes then sterilized using a pressure cooker for 15 minutes. After sterilization, the test tubes were left to cool and stored in a cool, dry place.

*Subculturing of the microorganism specimens*

The microbes used included *Bacillus cereus* and *Staphylococcus aureus* as the gram-positive bacteria, *Escherichia coli*, and *Pseudomonas aeruginosa* as the gram-negative bacteria, and *Candida albicans* as the fungus. Two loopful of each microbe were streaked using a sterilized wire loop on Trypton Soy Agar (TSA) plates and incubated for 24 hours at 37°C apart from *Candida albicans* which was incubated at room temperature 24.4°C. After that, the cultures were suspended in 10 ml of sterile physiological saline to achieve a concentration of 0.5 MacFarland Standards.

*Micro broth dilution*

The method described by the Clinical and Laboratory Standards Institute (CLSI) ^18^ was used. 3.2 g of the acetone, methanol and aqueous extracts were dissolved in 4 ml of MHB to achieve a concentration of 800 mg/ml. A series of 8 culture tubes containing 2 ml of MHB were lined up in a rack in duplicates per extract. Two-fold serial dilution was done from the stock solution and the concentrations achieved were 800 mg/ml, 400 mg/ml, 200 mg/ml, 100 mg/ml, 50 mg/ml, 25 mg/ml, 12.5 mg/ml, 6.25 mg/ml and 3.125 mg/ml. The tubes were then inoculated with 100 μl of the respective bacterial and fungal suspension using a 1 ml micropipette and incubated overnight at 35°C except for the fungal samples, which were kept at room temperature 24.4°C for 72 hours. A positive control was done where the test tubes included the microorganisms and commercially available antimicrobials. The antimicrobials used included ciprofloxacin (HIMEDIA SD142-ICT), erythromycin (HIMEDIA SD013-ICT), and fluconazole 150 mg capsule (Universal Corp). Dimethyl sulphoxide (DMSO) (Loba Chemie PVT LTD No 65530) was used as the negative control.

*Agar well diffusion*

The procedure recommended by the Clinical and Laboratory Standards Institute (CLSI) ^18^ was used. Mueller Hinton Agar (MHA) (OXOIDREF CMO337) was prepared by suspending 38g of the powder in 1 litre of purified water, mixed thoroughly, heat with frequent agitation, boiled for 1 minute to completely dissolve the powder and sterilized at 121°C for 15 minutes. The MHA was then poured onto sterilized petri dishes. The petri dishes were later inoculated with the respective microorganisms using cotton swabs. 4 wells of about 6 mm in diameter were punched aseptically using a sterile cork borer in each petri dish and numbered. 100 μl of the plant extract solution at different desired concentrations was added into the numbered wells. The plates were incubated at 37°C for 24 hours except those containing the fungal strain, which was incubated at room temperature 24.4°C. A positive control containing the respective antimicrobials and a negative control containing dimethyl sulphoxide (DMSO) (Loba Chemie, PVT LTD No 65530) were maintained in this experiment. The diameter of the zone of inhibition was measured in mm, which was used to determine microbial growth.Zones of inhibition diameter of ≤6.00 mm was interpreted as no activity and >6.00 mm as susceptible to the plant extracts.

*Determination of the minimum inhibitory concentration (MIC) and the minimum bactericidal concentration (MBC)*

The test tubes with the least concentration that did not record any growth or show turbidity from the micro broth dilution were recorded as the minimum inhibitory concentration (MIC) and were cultured on MHA petri dishes through the pour plate method and incubated overnight at 35°C. The lowest concentration with no visible growth was defined as the minimum bactericidal concentration (MBC), indicating 99.5% kill of the original inoculum.

### Sub-acute toxicity

In total, 20 male and 20 female *Sprague Dawley* rats (8-10 weeks old; 150-180 g) were sourced from the animal holding unit of the Department of Public Health, Pharmacology, and Toxicology, University of Nairobi, and labelled from 1-5 in such a way that there were 5 male and female animals bearing each number. The number assigned to each animal was written on a piece of paper, folded, and placed on a receptacle which was shaken. A paper was withdrawn at random and the animal which bore each of the numbers 1-5 was assigned into separate polypropylene cages (421×290×190 mm) which were labelled distilled water control group (group 1), low dose group (250 mg/kg or group 2), intermediate dose group (500 mg/kg or group 3), and high dose group (1000 mg/kg or group). Water and rat pellets (Unga Feeds) were provided to the animals *ad libitum.* The animals were fasted overnight after a 10-day period was allowed for the animals to acclimatize to the laboratory conditions. Individual dosing was based on the calculations described by Ochola and colleagues as below;

$$\text{Volume of extract required}\;\left(\mathrm{mL}\right)=\frac{\text{Dosage of extract}\left(\mathrm{mg}/\mathrm{kg}\right)\times \text{weight of}\;\mathrm{rat}\left(\mathrm{kg}\right)}{\text{Concentration}\left(\mathrm{mg}/L\right)}$$

The aqueous extract was administered once daily through intragastric gavage using a curved gavage needle to the animals in the high dose, intermediate dose, and low dose groups and then monitored for any behavioural, morbidity, and mortality changes. The control group received distilled water as the placebo. The weight was measured weekly and the dosing adjusted according to the weight of the rats. Approximately 200 g of rat’s pellets and 500 ml of water were measured and initially given to each cage. This was monitored and adjusted daily, and the consumption was recorded weekly. The temperature and humidity of the animal room where the animals were kept were recorded daily. Confounders were not controlled. All authors were aware of the group allocation at the different stages of the experiment, the outcome assessment, and the data analysis.

*Blood collection and organ harvesting*

After the 28 days study, the animals were monitored for an extra day in case of any delayed changes. They were fasted overnight, weighed, and anaesthetized using diethyl ether for blood collection through the retro-orbital vein using capillary tubes. Two sets of blood samples were collected. One set for the hematological assay was collected in Ethylene Diamine Tetra acetic acid (EDTA) tubes. The other set for the biochemistry assay was collected in clot activator tubes. The animals were humanely sacrificed by euthanasia using diethyl ether (FINAR Batch No. 70580LM500) in a glass chamber (long considered a humane method of sacrificing experimental animals such as rats), macroscopically observed for signs of gross lesions, and organs harvested. The organs collected included the liver, lungs, kidney, heart, and gonads.

*Hematological assay*

Blood samples collected in EDTA tubes were transported to Mama Lucy Teaching and Referral Hospital, where they were analyzed using a Swelab haematology analyzer (Alfa plus sampler BD AR product code. 1420046). The parameters examined included hemoglobin (HGB), red blood cells (RBC), white blood cells (WBC), platelets (PLT), lymphocytes (LYM), mean corpuscular hemoglobin concentration (MCHC), and mean corpuscular volume (MCV).

*Biochemical assay*

Blood samples collected in the clot activator tubes were left to stand for 15 minutes to allow time for clotting. They were later centrifuged for 15 minutes to obtain the supernatant. A Pasteur pipette was used to transfer the serum into Eppendorf tubes, and these were transported to Embu Level 5 Hospital in an icebox. The analysis was done using a Humastar 100 analyzer (REF 16890). The parameters examined included urea, creatinine, total bilirubin, direct bilirubin, alkaline phosphatase, alanine aminotransferase, aspartate aminotransferase, total protein, and albumin.

*Histopathology*

After necropsy, the organs harvested were preserved in containers containing 10% formalin and transported to the histology laboratory for analysis. Biopsies were obtained, fixed in 10% neutral formalin, processed, and embedded in paraffin wax. Tissue blocks of 5 microns (5 μ) were sectioned using a microtome, stained with hematoxylin and eosin stain, and observed under a light microscope (Olympus CX21FS1. Magnification ×400).

*Disposal of carcasses*

The rat carcasses were put in red biohazard bags and disposed of in the veterinary pathology disposal pit according to Biosafety, Animal Care, and Use Committee guidelines of the University of Nairobi.

### Data analysis

Antimicrobial efficacy data were summarized in tables. Data on hematological and biochemical parameters were summarized in MS Excel (2016), exported into GenStat statistical software (Version 15, RRID:SCR_014595), and analyzed using one-way analysis of variance (ANOVA) and Tukey’s multiple comparisons test. *p<0.05* was considered significant.

## Results

All the herbalists interviewed in Embu County were male ( Table 1). ^44^ Most herbalists were married, >50 years old, had a Primary level of education, had practiced between 11 and 20 years, did not receive any formal training, and came from Kithunthuri sub-location.

**Table 1.** Demographic characteristics of herbalists interviewed in Embu County (n=35).

Variable (n=35)	Frequency (percentage)
**Sex**	
Male	35 (100.0)
Female	0 (0.0)
**Marital status**	
Single	3 (8.6)
Married	31 (88.6)
Not specified	1 (2.9)
**Age (years)**	
18-34	1 (2.9)
35-49	7 (20)
>50	27 (77.1)
**Level of education**	
Illiterate	8 (22.9)
Primary (grade 1-8)	21 (60)
Secondary (junior and senior high school)	6 (17.1)
**Years of herbal practice**	
0-5	2 (5.7)
6-10	11 (31.4)
11-20	12 (34.3)
>20	6 (17.1)
Not captured	4 (11.4)
**Received formal training on herbal medicine**	
Yes	3 (8.6)
No	32 (91.4)
**Sub location**	
Kithunthuri	9 (25.7)
Gacegithiuri	6 (17.1)
Nyangwa	4 (11.4)
Kianjiru	3 (8.6)
Mbita	3 (8.6)
Gikiiro	2 (5.7)
Kirima	1 (2.9)
Machangia	1 (2.9)
Mariari	1 (2.9)
Mavuria	1 (2.9)
Mombo munyiri	1 (2.9)
Mulindi	1 (2.9)
Mutuobare	1 (2.9)
Tigoo	1 (2.9)

Table 2 shows the local names of the plants, preparation, dosage and management of side effects.

**Table 2.** Local names, parts used, sources, indications, preparation, frequency of use, dosage and duration, side effects, and storage of *Catha edulis* (Vahl.) Endl.

**Local names of the plant**	Miraa, Muguka, Mugombe, Mutimutiri, Mugumo, Mutamucii, Muraa, Mugwathingi, Gitune, Mwirugi, Gituu, Karuki, Muruti, Mutamuai
**Parts used**	Leaves, roots
**Source of the plant**	Local/personal farms; Kiritini, Mbeere south, Nyangwa, Kerwa, Gikondi, and Gacegethiuri
**Human indications**	Cough, fatigue, fever, stomachache, heartburn, diarrhoea, chest congestion, constipation, and stress relief
**Veterinary indications**	Constipation, diarrhoea (goats), stomachache, and constipation (cows and goats)
**Preparation**	Chew fresh leaves or maceration of the leaves with water and use the filtrate
**Frequency of use**	When necessary, once, twice, or thrice
**Dosage and duration**	**Fresh leaves**: two handfuls, when necessary, 5-20 leaves, ten leaves ground into powder, 30 leaves **Liquid:** 50 ml for two days, 250 ml until diarrhoea stops, 50 mls+2 drops of Aloe Vera for three days, 50 ml for three days, 200 ml for three days, and 30 ml for two days **Syringe**: 10 ml or 20 ml **Tablespoonful**: Once daily for five days, two tablespoonfuls for three days, and two tablespoonfuls for five days **Glass/cup**: one glass of crude preparation, one cup for five days, ¼ of a glass for three days, 100 ml using a measuring cup, and ½ a glass for three days **Teaspoonful:** two teaspoonfuls for four days or two teaspoonfuls for five days
**Side effects**	Constipation, ulcers, discoloration of teeth, and cracking of teeth
**Management of side effects**	Ulcers (Kiathaa is used), constipation (Kithee is used), patients are advised to brush their teeth regularly or after chewing the leaves, eat cabbage, or drink plenty of water
**Management of addiction**	Clients are advised to reduce intake, stop medication after the treatment period, they were observed closely and were advised to have self-discipline.
**Storage**	The preparations in plastic bottles were kept in a cool, dry place or kept in a cupboard. The fresh leaves were stored in opaque bottles, polythene bags, and woven bags and were wrapped using newspapers. The leaves were also mixed with lemon to avoid spoilage. In general, all the preparations in plastic bottles and fresh leaves were kept away from direct sunlight.

Table 3 summarizes the antimicrobial activity of different solvent extracts of *Catha edulis* against common gram+ve, gram-ve, and fungal microbes. ^45^

**Table 3.** Antimicrobial properties of *Catha edulis* against gram-positive, gram-negative, and fungal microorganisms.

Microorganism	Zone of inhibition (mm)					
	Concentration (mg/mL)	Acetone extract	Aqueous extract	Methanol extract	Positive control	Negative control
					Ciprofloxacin	Dimethylsulfoxide
***Bacillus cereus***	3.125	6.00±0.00	6.00±0.00	6.00±0.00	32.00±0.00	0.00±0.00
	6.25	6.00±0.00	6.00±0.00	6.00±0.00		
	12.50	6.00±0.00	6.50±0.71	6.00±0.00		
	25.00	8.50±3.54	8.00±0.00	6.00±0.00		
	50.00	12.00±4.24	9.00±0.00	9.00±0.00		
	100.00	15.00±8.49	11.50±0.71	12.00±0.00		
	200.00	12.00±2.83	12.50±0.71	12.50±0.71		
	400.00	14.00±1.41	14.50±0.71	15.50±0.71		
	800.00	18.00±1.41	16.00±0.00	17.00±0.00		
***Staphylococcus aureus***	3.125	6.00±0.00	6.00±0.00	6.00±0.00	32.00±0.00	0.00±0.00
	6.25	6.00±0.00	6.00±0.00	6.00±0.00		
	12.50	6.00±0.00	6.00±0.00	6.00±0.00		
	25.00	7.50±0.71	6.00±0.00	6.00±0.00		
	50.00	7.50±0.71	6.00±0.00	7.50±0.71		
	100.00	8.50±2.12	6.50±0.71	9.50±0.71		
	200.00	9.50±0.71	7.50±0.71	10.50±0.71		
	400.00	14.50±0.71	11.00±0.00	11.00±1.41		
	800.00	15.00±0.00	16.00±0.00	18.00±1.41		
					**Erythromycin**	
***Pseudomonas aeruginosa***	800	6.00±0.00	6.00±0.00	6.00±0.00	15.00±0.00	6.00±0.00
***Escherichia coli***	800	6.00±0.00	6.00±0.00	6.00±0.00	14.50±0.71	6.00±0.00
					**Fluconazole**	
***Candida albicans***	800	6.00±0.00	60.00±0.00	6.00±0.00	56.50±0.71	6.00±0.00

Table 4 summarizes the effect of varying doses of the aqueous extract of *Catha edulis* on various hematological parameters in male rats. ^46^

**Table 4.** Effects of the aqueous extract of *Catha edulis* on the hematological parameters of male *Sprague Dawley* rats.

Treatment (n=5)	WBC 10 ^9^/L	LYM 10 ^9^/L	HGB g/dL	MCHC g/dL	RBC 10 ^12^/L	MCV fL	PLT 10 ^9^/L
Control	11.06±1.79 ^a^	7.97±1.53 ^a^	15.84±0.73 ^a^	35.72±0.85 ^a^	7.89±0.46 ^a^	56.28±2.43 ^a^	581.40±94.08 ^a^
250 mg/kg	13.40±5.48 ^a^	9.92±4.94 ^a^	15.56±2.04 ^a^	35.86±1.15 ^a^	7.59±0.93 ^a^	57.20±2.84 ^a^	539.40±186.10 ^a^
500 mg/kg	13.74±4.29 ^a^	11.42±4.16 ^a^	15.02±0.90 ^a^	34.92±0.70 ^a^	7.52±0.64 ^a^	57.50±3.63 ^a^	549.20±95.92 ^a^
1000 mg/kg	11.84±4.87 ^a^	9.34±4.94 ^a^	14.98±1.12 ^a^	34.96±0.67 ^a^	7.35±0.78 ^a^	58.56±3.23 ^a^	475.20±97.00 ^a^

WBC: White blood cells; LYM: Lymphocytes; HGB: Hemoglobin; MCHC; Mean corpuscular hemoglobin concentration; RBC: Red blood cells; MCV; Mean corpuscular volume; PLT; platelets. Means with different subscript along the columns are significantly different at *p<0.05*.

There was no significant difference ( *p*>0.05) between the mean WBC, LYM, HGB, MCHC, RBC, MCV, and PLT levels in male rats who received a 250 mg/kg dose of the aqueous extract of *Catha edulis* and the mean WBC, LYM, HGB, MCHC, RBC, MCV, and PLT levels in male rats who received distilled water. There was no significant difference ( *p*>0.05) between the mean WBC, LYM, HGB, MCHC, RBC, MCV, and PLT levels in male rats who received a 500 mg/kg dose of the aqueous extract of *Catha edulis* and the mean WBC, LYM, HGB, MCHC, RBC, MCV, and PLT levels in male rats who received distilled water ( Table 4). There was no significant difference ( *p*>0.05) between the mean WBC, LYM, HGB, MCHC, RBC, MCV, and PLT levels in male rats who received a 1000 mg/kg dose of the aqueous extract of *Catha edulis* and the mean WBC, LYM, HGB, MCHC, RBC, MCV, and PLT levels in male rats who received distilled water ( Table 4).

Table 5 summarizes the effect of varying doses of the aqueous extract of *Catha edulis* on various hematological parameters in female rats.

**Table 5.** Effects of the aqueous extract of *Catha edulis* on hematological parameters of female *Sprague Dawley* rats.

Treatment (n=5)	WBC 10 ^9^/L	LYM 10 ^9^/L	HGB g/dL	MCHC g/dL	RBC 10 ^12^/L	MCV fL	PLT 10 ^9^/L
Control	9.04±3.39 ^a^	6.56±2.60 ^a^	13.58±2.32 ^a^	39.10±0.52 ^a^	6.28±1.13 ^a^	55.46±1.78 ^a^	497.20±121.10 ^a^
250 mg/kg	8.74±2.77 ^a^	6.68±2.06 ^a^	15.60±0.57 ^a^	33.90±7.32 ^a^	7.45±0.46 ^ab^	55.58±2.30 ^a^	607.40±97.80 ^a^
500 mg/kg	8.14±1.59 ^a^	6.14±1.08 ^a^	15.58±0.45 ^a^	36.82±0.43 ^a^	7.53±0.18 ^b^	56.16±0.88 ^a^	559.20±107.20 ^a^
1000 mg/kg	7.18±1.59 ^a^	5.23±1.00 ^a^	16.05±0.52 ^a^	37.48±0.69 ^a^	7.78±0.48 ^b^	55.10±2.17 ^a^	634.60±21.31 ^a^

WBC: White blood cells; LYM: Lymphocytes; HGB: Hemoglobin; MCHC; Mean corpuscular hemoglobin concentration; RBC: Red blood cells; MCV; Mean corpuscular volume; PLT; platelets. Means with different subscript along the columns are significantly different at *p<0.05*.

There was no significant difference ( *p*>0.05) between the mean WBC, LYM, HGB, MCHC, RBC, MCV, and PLT levels in female rats who received a 250 mg/kg dose of the aqueous extract of *Catha edulis* and the mean WBC, LYM, HGB, MCHC, RBC, MCV, and PLT levels in female rats who received distilled water ( Table 5). There was no significant difference ( *p*>0.05) between the mean WBC, LYM, HGB, MCHC, RBC, MCV, and PLT levels in female rats who received a 500 mg/kg dose of the aqueous extract of *Catha edulis* and the mean WBC, LYM, HGB, MCHC, RBC, MCV, and PLT levels in female rats who received distilled water ( Table 5). There was no significant difference ( *p*>0.05) between the mean WBC, LYM, HGB, MCHC, RBC, MCV, and PLT levels in female rats who received a 1000 mg/kg dose of the aqueous extract of *Catha edulis* and the mean WBC, LYM, HGB, MCHC, RBC, MCV, and PLT levels in female rats who received distilled water ( Table 5).

Table 6 summarizes the effect of varying doses of *Catha edulis* on the biochemical parameters in male rats.

**Table 6.** Effect of the aqueous extract of *Catha edulis* on biochemical parameters in male rats.

Treatment	Albumin g/L	ALP U/I	ALT U/I	AST U/I	Creatinine μmol/L	Direct Bilirubin μmol/L	Total Bilirubin μmol/L	Total Protein g/L	Urea mmol/L
Control	3.80±0.19 ^a^	265.40±59.34 ^a^	111.40±24.99 ^a^	210.60±42.77 ^a^	35.41±5.44 ^a^	0.68±0.46 ^a^	11.82±6.13 ^a^	70.40±4.49 ^a^	8.75±0.64 ^a^
250 mg/kg	3.73±0.32 ^a^	427.80±240.10 ^a^	118.20±15.53 ^a^	231.60±17.67 ^a^	39.29±4.84 ^a^	1.13±0.12 ^a^	8.45±3.83 ^a^	71.85±5.27 ^a^	8.28±1.27 ^a^
500 mg/kg	3.76±0.11 ^a^	349.40±127.70 ^a^	120.00±17.07 ^a^	257.80±39.79 ^a^	40.83±5.02 ^a^	1.24±0.56 ^a^	15.47±10.69 ^a^	73.13±3.43 ^a^	9.34±1.13 ^a^
1000 mg/kg	3.73±0.19 ^a^	473.20±150.60 ^a^	125.60±13.13 ^a^	254.80±35.49 ^a^	39.12±2.57 ^a^	1.92±1.40 ^a^	16.41±11.34 ^a^	72.66±4.54 ^a^	8.39±1.35 ^a^

ALP; Alkaline phosphatase; ALT: Alanine amino transferase; AST; Aspartate amino transferase. Means with different superscripts along the columns are significantly different at *p<0.05.*

There was no significant difference ( *p*>0.05) between the mean albumin, ALP, ALT, AST, creatinine, direct bilirubin, total bilirubin, total protein, and urea levels in male rats who received a 250 mg/kg dose of the aqueous extract of *Catha edulis* and the mean albumin, ALP, ALT, AST, creatinine, direct bilirubin, total bilirubin, total protein, and urea levels in male rats who received distilled water ( Table 6). There was no significant difference ( *p*>0.05) in the mean albumin, ALP, ALT, AST, creatinine, direct bilirubin, total bilirubin, total protein, and urea levels in male rats who received a 500 mg/kg dose of the aqueous extract of *Catha edulis* and the mean albumin, ALP, ALT, AST, creatinine, direct bilirubin, total bilirubin, total protein, and urea levels in male rats who received distilled water ( Table 6). There was no significant difference ( *p*>0.05) in the mean albumin, ALP, ALT, AST, creatinine, direct bilirubin, total bilirubin, total protein, and urea levels in male rats who received a 1000 mg/kg dose of the aqueous extract of *Catha edulis* and the mean albumin, ALP, ALT, AST, creatinine, direct bilirubin, total bilirubin, total protein, and urea levels in male rats who received distilled water ( Table 6).

Table 7 summarizes the effect of varying doses of *Catha edulis* on the biochemical parameters in female rats. ^47^

**Table 7.** Effect of the aqueous extract of *Catha edulis* on biochemical parameters in female rats.

Treatment	Albumin g/L	ALP U/I	ALT U/I	AST U/I	Creatinine μmol/L	Direct Bilirubin μmol/L	Total Bilirubin μmol/L	Total Protein g/L	Urea mmol/L
Control	3.97±0.14 ^a^	106.60±39.70 ^a^	90.61±12.68 ^a^	179.20±29.52 ^a^	42.02±4.21 ^a^	1.27±0.47 ^a^	11.98±4.00 ^ab^	69.72±1.52 ^a^	7.17±0.74 ^a^
250 mg/kg	3.91±0.12 ^a^	140.00±52.60 ^a^	87.01±11.81 ^a^	197.20±38.57 ^a^	42.41±3.63 ^a^	1.38±0.23 ^a^	8.61±1.33 ^a^	68.77±2.84 ^a^	7.48±0.84 ^ab^
500 mg/kg	3.80±0.50 ^a^	209.60±229.40 ^a^	94.01±16.37 ^a^	215.60±51.91 ^a^	40.83±3.63 ^a^	1.22±0.15 ^a^	15.15±3.93 ^ab^	70.81±3.65 ^a^	9.35±1.72 ^b^
1000 mg/kg	3.89±0.12 ^a^	143.50±46.08 ^a^	94.51±14.80 ^a^	231.50±20.27 ^a^	36.16±1.42 ^a^	1.92±1.15 ^a^	18.95±8.47 ^b^	72.02±2.35 ^a^	8.76±0.79 ^ab^

ALP; Alkaline phosphatase; ALT: Alanine amino transferase; AST; Aspartate amino transferase. Means with different superscripts along the columns are significantly different at *p<0.05.*

There was no significant difference ( *p*>0.05) between the mean albumin, ALP, ALT, AST, creatinine, direct bilirubin, total protein, total bilirubin, and urea levels in female rats who received a 250 mg/kg dose of the aqueous extract of *Catha edulis* and the mean albumin, ALP, ALT, AST, creatinine, direct bilirubin, total protein, total bilirubin, and urea levels in female rats who received distilled water. There was no significant difference ( *p*>0.05) between the mean albumin, ALP, ALT, AST, creatinine, direct bilirubin, total protein, total bilirubin and urea levels in female rats who received a 500 mg/kg dose of the aqueous extract of *Catha edulis* and the mean albumin, ALP, ALT, AST, creatinine, direct bilirubin, total protein, total bilirubin and urea levels in female rats who received distilled water ( Table 7). There was no significant difference ( *p*>0.05) between the mean albumin, ALP, ALT, AST, creatinine, direct bilirubin, total protein, total bilirubin and urea levels in female rats who received a 1000 mg/kg dose of the aqueous extract of *Catha edulis* and the mean albumin, ALP, ALT, AST, creatinine, direct bilirubin, total protein, total bilirubin and urea levels in female rats who received distilled water ( Table 7).

**Figure 2.**
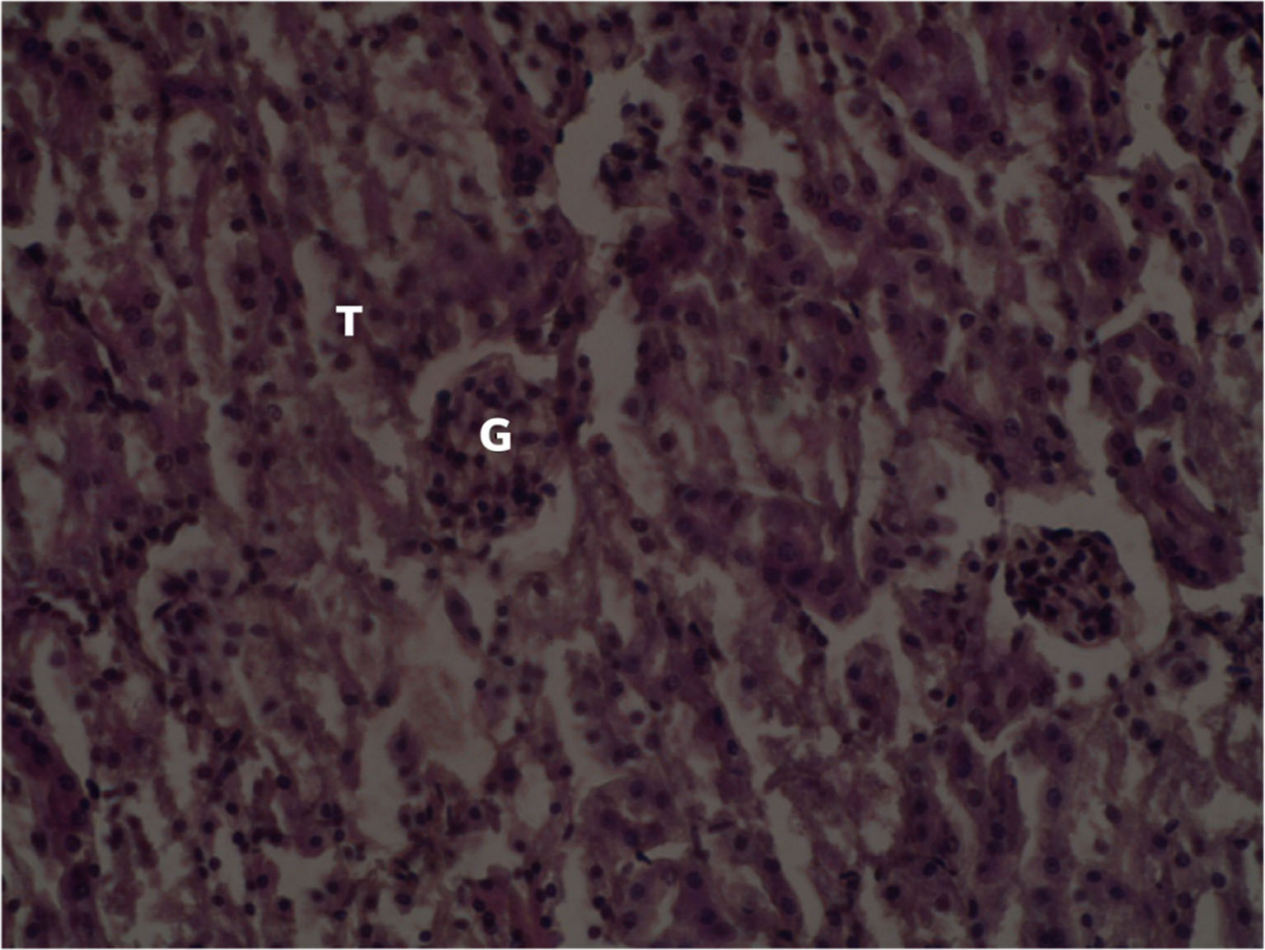
Normal renal parenchyma of the control group (×400). No adjustments in terms of contrast or brightness. T: Renal tubules G: Glomerulus

Figure 2 summarizes the effects of distilled water on the histological section of the kidney of a female *Sprague Dawley* rat treated with distilled water.

**Figure 3.**
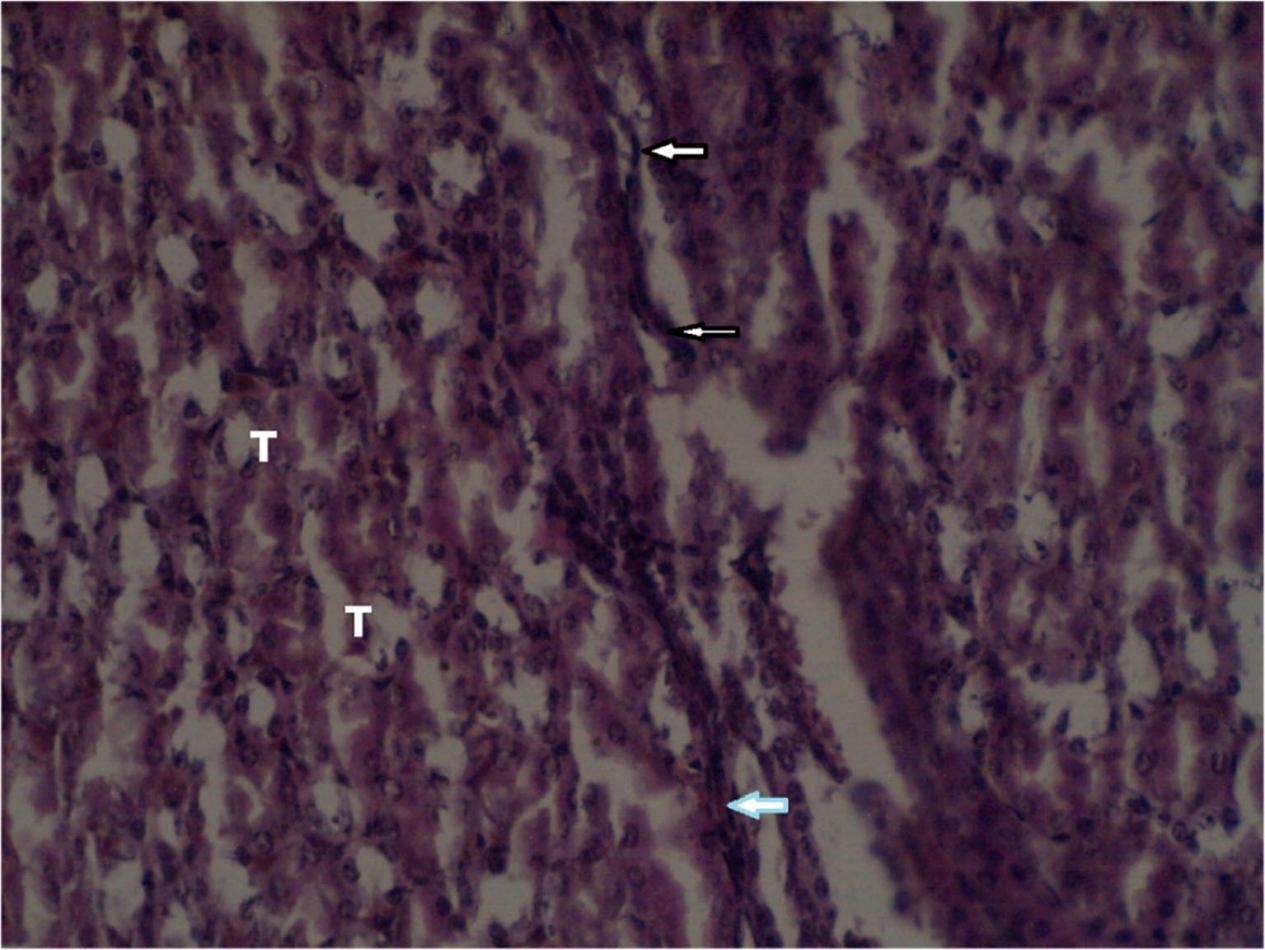
Localized interstitial connective tissue proliferation shown by the arrow indicating multifocal kidney interstitial hemorrhage in the intermediate dose group (×400). No adjustments were made in terms of contrast or brightness.

**Figure 4.**
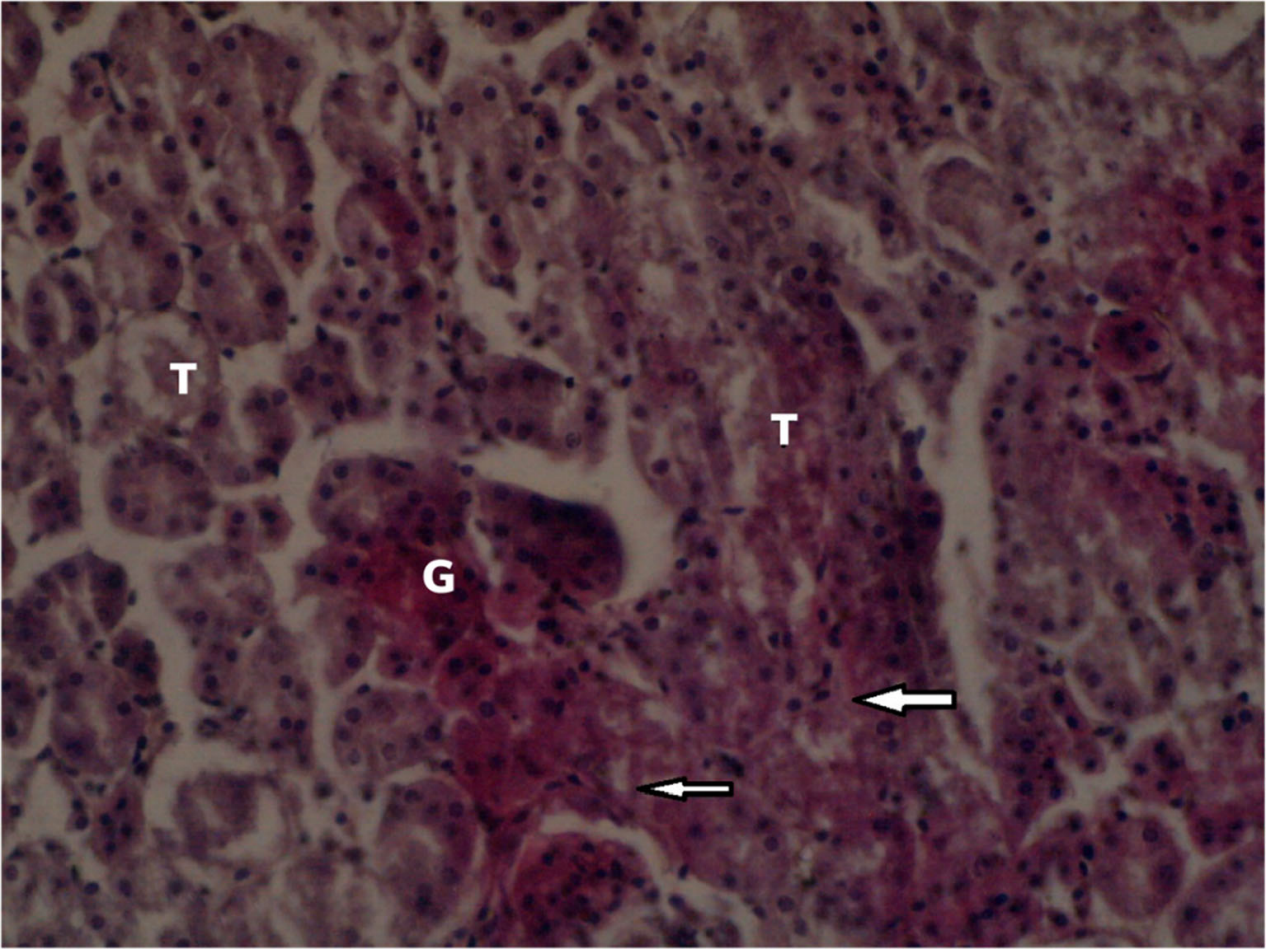
Localized tubular epithelium necrosis in the low dose group (×400). No adjustments were made in terms of contrast or brightness.

Figure 3 summarizes the effects of an intermediate dose of *Catha edulis* (Vahl.) Endl. on the histological section of the kidney of a female *Sprague Dawley* rat. The effects of a low dose of *Catha edulis* on the histological section of the kidney in the female Sprague Dawley rat is shown in Figure 4. All data from the rats and the raw microscope images can be found as *Underlying data.* ^48^^,^^49^

## Discussion

### Ethnopharmacological uses of *Catha edulis* (Vahl.) Endl.

Studies on the ethnomedicinal value of *Catha edulis* (Vahl.) Endl are scarce. Kiunga and colleagues conducted a survey of traditional medicinal uses of *Catha edulis* in Meru and Embu Counties of Kenya between September 2014 and February 2015 and reported that most of the informants in the study were males (32/42; 76.19%), aged 45-84 years, and had a low level of education. ^17^ These observations compare well with the present study’s findings, where all 35 informants interviewed were males. Most were aged above 50 years and had only a primary level of education. These observations in both studies are not surprising considering that the cultivation and use of *Catha edulis* is mainly a preserve of men in African and Arabian societies. ^10^^,^^17^^,^^19^ Other findings of Kiunga and colleagues were that the leaves of *Catha edulis* were the main parts used, and the results of the present study bear some similarity since leaves and roots were the main parts used. Kiunga *et al*. also reported that chewing fresh material was the primary method of crude drug preparation. ^17^ This is similar to the observations of the present study, where the informants reported that the medication from *Catha edulis* was primarily prepared by chewing fresh leaves or macerating the leaves with water and using the filtrate. Diarrhoea, gonorrhea, and toothache were the main indications of *Catha edulis.* Treatment was initiated after fresh plant material was boiled as per Kiunga and colleagues. ^17^ In contrast, the present study delineated human and veterinary indications of *Catha edulis.* Some of the indications in the present study overlap those of Kiunga *et al*., but more indications are provided in the present study, including stomachache, cough, fatigue, fever, heartburn, chest congestion, constipation, and stress relief. Kiunga *et al*. identified 11 Traditional varieties of *Catha edulis.* ^17^ This study observed that the local people had 14 different ways to call *Catha edulis.* Some of the varieties identified in the survey by Kiunga *et al*. were also observed in the present study and included *Gituu, Muguka, Mugumo, Mugwathingi, Muti-mutiri.* Kiunga and colleagues observed that *Catha edulis* leaves were primarily administered orally, there was no proper dosage for any given ailment, and specific varieties had adverse health effects. ^17^ In the present study, preparations of *Catha edulis* were primarily administered orally, and the dosages, frequency, and duration of use were spelt out. Still, there was no uniformity in the treatment regimens. The storage condition of the preparations, the side effects, and their management was also observed. In summary, the observations of the present study contribute to the body of knowledge on *Catha edulis* documented previously by Kiunga and colleagues, particularly as far as the plant parts which were used, preparation of crude drugs, indications, varieties of *Catha edulis*, dosages, frequency, the duration of use, side effects and their management.

### Antimicrobial properties of *Catha edulis*

The aqueous, acetone, and methanol extracts of *Catha edulis* were ineffective against *Escherichia coli, Pseudomonas aeruginosa*, and *Candida albicans.* Moreover, the extracts had limited efficacy against *Bacillus cereus* and *Staphylococcus aureus* relative to the standard antimicrobial agents. Kithinji *et al*. evaluated the antimicrobial properties of the aqueous twig and leaf extracts of *Catha edulis* from Ithanja village in Meru County against *Staphylococcus aureus, Streptococcus pyogenes, Streptococcus pneumoniae, Escherichia coli,* and *Candida albicans* and reported that all the tested concentrations significantly inhibited the growth of all bacterial pathogens except *Escherichia coli* and was ineffective against *Candida albicans.* ^14^ Siddiqui *et al*. evaluated the antibacterial and anti-acanthamoebic properties of the crude methanolic leaf and shoot extracts of *Catha edulis* procured from a Somali shop on Edgware Road, London, UK. ^19^ Their study established that the crude methanolic leaf and shoot extracts of *Catha edulis* were amoebicidal against *Acanthamoeba castellanii*, had potent antibacterial activity against *Brevundimonas diminuta, Bacillus magaterium,* and *Micrococcus luteus.* ^19^ However, it was ineffective against *Escherichia coli* and yeast ( *Aspergillus varicoluor, Penicillum solitum*, and *Penicillum brevicompactum*). ^19^ Limited sensitivity of *Porphyromonas gingivalis* and *Tannerella forsythensis* and lack of sensitivity of *Veillonella parvula, Actinomyces israelii,* and some Streptococci to the aqueous extracts of *Catha edulis* prepared from three cultivars in Yemen has also been reported. ^16^ Moreover, Fatima and co-workers evaluated the GC-MS phytochemical profile and antimicrobial properties of aqueous, methanol, and dimethyl sulfoxide (DMSO) extracts of *Catha edulis* cultivated in Saudi Arabia. They reported that these extracts had good antimicrobial activity against *Staphylococcus aureus, Streptococcus pyogenes, Escherichia coli, Klebsiellae pneumoniae, Proteus mirabilis, Pseudomonas aeruginosa,* and *Candida albicans.* ^20^

Gram-negative bacteria such as *Escherichia* coli and *Pseudomonas aeruginosa* have complex cell walls which comprise peptidoglycan and an outer membrane of lipopolysaccharides and lipoproteins. ^21^^,^^22^ It could be argued that this complex cell may have limited the capacity of the acetone, aqueous, and methanol extracts of *Catha edulis* to interact with critical intracellular components of the bacteria. ^22^^,^^23^ The ineffectiveness of the acetone, aqueous, and methanol leaf extracts of *Catha edulis* towards *Candida albicans* may have something to do with chitin on the fungus cell wall, which contributes to the cell wall strength and stability, thereby impairing the penetration of the prepared extracts. ^22^^,^^24^ The ineffectiveness of the extracts towards *P. aeruginosa* may be due to the inability of the prepared extracts to interfere with the permeability of the cytoplasmic membrane of the pathogen. ^22^^,^^25^

### Toxicity of *Catha edulis*

Estimates suggest that up to 20 million people chew *Catha edulis* daily and many more rely on the plant as a source of livelihood. ^4^^,^^7^ Although such a vast population of people is exposed to *Catha edulis* habituation, very few studies are available in the literature to shed light on its toxicity. Therefore, it is essential to evaluate the toxicological properties of *Catha edulis* in suitable animal models.

The present investigation used a 28-day repeated dose experimental design to evaluate the effects of different doses (250 mg/kg bwt, 500 mg/kg bwt, 1000 mg/kg bwt) of the aqueous leaf extract of *Catha edulis* on organ histology, biochemical, and haematological parameters of *Sprague Dawley* rats (female and male). The results of toxicity evaluation of the aqueous leaf extracts of *Catha edulis* were a mixed bag. Many of the parameters evaluated suggested that the extract was safe, but a few of the parameters may be a cause of concern. Female and male *Sprague Dawley* rats that received the aqueous leaf extract of *Catha edulis* exhibited a non-significant reduction in food intake after the first seven days of treatment. Similar observations were made by Al-Mamary and colleagues, except that the animals studied (New Zealand White Rabbits) regained their appetite. ^15^ There was no significant decrease in the water intake of female and male rats in the second week of treatment. A significant reduction in the weight of female rats that received the highest dose (1000 mg/kg bwt) of the aqueous leaf extract of *Catha edulis* was observed between week one and week two and between week one and week three. Decreased food and water intake do not seem to cause of weight loss, suggesting that the plant could potentially have some anti-obesity effects. A decrease in body weight could be related to leptin levels. ^26^ Al-Shaggha and colleagues carried out a scoping review of animal and human studies on *Catha edulis.* They concluded that there was acceptable evidence suggesting that plant extracts produced changes in weight, fat mass, appetite, lipid biochemistry, and hormonal levels in humans and animals. ^27^ It was further reported that the differences were more pronounced at higher doses and longer durations of interventions. ^27^ The observation that female rats were more vulnerable to weight loss than their male counterparts agrees with OECD guidelines. ^28^

Blood is a constantly circulating fluid that provides nutrition and oxygen and offers waste removal. Therefore, it is a focal point of exposure to foreign substances, which may have untoward effects. ^29^^,^^30^ By analyzing the different components of blood, it may be possible to have a sense of the physiological and pathological status of the body. ^29^ Haematological analysis in the case of this study suggested that there were no significant differences in the WBC, LYM, HGB, MCHC, MCV, and PLT levels in male and female rats treated with graded doses of *Catha edulis* relative to the rats in the control group. However, it was observed that the mean levels of RBC in female rats treated with the highest dose of *Catha edulis* were significantly higher than the mean levels in the control group. This was contrary to the findings of Ismaeel *et al*. ^31^ They found out that rats who received *Catha edulis* hydro-ethanol extract developed a reduction in RBC count compared to the control rats who received normal saline. This variation could be due to the dose administered and the type of extracts used. A similar finding was identified by Alele and colleagues whereby they suggested the polycythaemia could be due to a decrease in plasma volume without change in RBC mass in which the erythrocytes become more concentrated. ^32^

Most xenobiotics that enter the body through the oral route are absorbed via the gastrointestinal tract, transported to the liver, and bio-transformed into polar, water-soluble, compounds which may be easily eliminated (excreted). ^33^^,^^34^ However, toxic chemicals may also be produced from the process. ^35^ Microsomal cytochrome P _450’s_ monooxygenases and other enzymes are located in the liver but may also be expressed in various extrahepatic tissues. ^36^^,^^37^ The renal proximal tubule is a primary target for xenobiotic-induced renal toxicity and is also a site where P _450’s_ are primarily expressed in the kidney. ^38^^,^^39^ Nephrotoxic metabolites may be produced locally through P _450_ activity or in the liver/other organ and transported to the kidney through blood circulation. ^40^ High renal blood flow and high concentrations of excretory products borne from the re-absorption of water from the tubular fluid are other vital factors in the susceptibility of the liver to xenobiotics. ^41^ Increases in the levels of liver enzymes are usually indicative of liver damage. ^29^^,^^42^ In the present study, there was no significant difference in the mean levels of ALP, ALT, AST, albumin, creatinine, direct bilirubin and total protein in male and female rats which received different doses of the aqueous leaf extracts of *Catha edulis.* This indicates that the extract is unlikely to be harmful to the liver. However, the mean levels of total bilirubin and urea in female rats who received a 1000 mg/kg dose of *Catha edulis* were significantly higher ( *p*<0.05) than the mean levels of rats in the control group. Bilirubin is a product of heme catabolism. It is conjugated with glucuronic acid in the liver, making it soluble before it is excreted in bile. ^43^ The bilirubin levels may be elevated in various disease states, making it a good indicator of liver damage. ^43^ The observed increase in serum total bilirubin and urea could suggest liver and kidney damage, respectively, but this is inconclusive as the other biomarkers were normal. ^26^

Histopathological examination of the liver, lung, kidney and heart of the female *Sprague Dawley* rats and testes of the male *Sprague Dawley* rats revealed high and intermediate doses of the aqueous leaf extracts of *Catha edulis* produced local congestion of hepatic vessels in female rats. Furthermore, a low dose of the aqueous extract of *Catha edulis* produced localized interstitial connective tissue proliferation, multifocal kidney interstitial haemorrhage, and localized tubular epithelium necrosis in the kidney of some female rats. On the other hand, the lungs and testes, showed no adverse effects. The histoarchitecture of the liver of female and male *Sprague Dawley* rats who received a high dose (2000mg/kg bwt) of *Catha edulis* leaf extract showed degenerative vacuolation, coagulative necrosis in the pericentral region, haemorrhage, and congestion. ^26^ Such changes were minimal in the medium dose (1000mg/kg bwt) group. ^26^ Moderate and severe necrotic lesions in the renal parenchyma were observed in female rats who received medium and high doses of *Catha edulis.* ^26^ The histopathological findings in this study suggest chronic renal injury in female rats who received intermediate and low doses of *Catha edulis.*

## Conclusions

Married males who were >50 years of age with low levels of education are the primary custodians of ethnopharmacological knowledge among the Mbeere South community of Embu County. There is a need to scientifically validate the traditional claims on *Catha edulis* emanated from the Mbeere South community of Embu County. Limited antimicrobial efficacy and toxicity at high doses limit the ethnopharmacological use of the leaves of *Catha edulis.*

## Data availability

### Underlying data

Figshare: Questionnaire data. https://doi.org/10.6084/m9.figshare.16970701.v1. ^44^

Figshare: Antimicrobial data_Kariuki_ *Catha edulis.* https://doi.org/10.6084/m9.figshare.19036946.v2. ^45^

Figshare: the haematological parameters in male and female rats treated with *Catha edulis* from Embu County. https://doi.org/10.6084/m9.figshare.19039025.v1. ^46^

Figshare: Raw data_Biochemistry_Kariuki. https://doi.org/10.6084/m9.figshare.19039607.v2. ^47^

Figshare: Raw data from all rats on all days. https://doi.org/10.6084/m9.figshare.19232397.v1. ^48^

Data are available under the terms of the Creative Commons Zero “No rights reserved” data waiver (CC0 1.0 Public domain dedication).

Figshare: Unedited microscope images. https://doi.org/10.6084/m9.figshare.19165028.v1. ^49^

Data are available under the terms of the Creative Commons Attribution 4.0 International license (CC-BY 4.0).

### Extended data

Figshare: Questionnaire. https://doi.org/10.6084/m9.figshare.16970701.v4. ^50^

### Reporting guidelines

Figshare: ARRIVE checklist for ‘Studies on the ethnopharmacology, antimicrobial activity, and toxicity of Catha edulis (Vahl.) Endl., in Sprague Dawley rats’. https://doi.org/10.6084/m9.figshare.19166408.v2. ^51^

Data are available under the terms of the Creative Commons Zero “No rights reserved” data waiver (CC0 1.0 Public domain dedication).
