# Supplementary material for: Studies on the ethnopharmacology, antimicrobial activity, and toxicity of Catha edulis (Vahl.) Endl., in Sprague Dawley rats
Source: F1000Res. 2022 Aug 18;11:286. Originally published 2022 Mar 7. [Version 2] doi: 10.12688/f1000research.109243.2 (PMC10521044; doi:10.12688/f1000research.109243.2)
Supplement: Peer review history

# Reviewer report: Reviewer response for version 1

**DOI:** 10.5256/f1000research.120721.r137384

**Published:** 2022-05-31

**Authors:** Julia Kimondo

**Affiliations:** 1 Department of Pharmacology & Pharmacognosy, Jomo Kenyatta University of Agriculture and Technology, Nairobi, Kenya

Some comments:

1. Add a few sentences hypothesizing about the causes of the observations on weight loss similar to what was written on the polycythemia. The writers mentioned that a decrease in body weight could be related to leptin level, can you find some data that relates cathine or amphetamine like drugs to leptin levels? Look for data that relates the active ingredients in Khat with cause of weight loss.
2. About the histopathological examinations, is there a relation with cathines/amphetamines in causing injury to some of these organs, and could these compounds in Khat be the cause?
3. The human indications seem to be contrary to your findings. Is it possible that these indications may have been brought about by stress and hence the contradictions? See how you can add a few sentences to the discussion about this.

Is the work clearly and accurately presented and does it cite the current literature?

Yes

If applicable, is the statistical analysis and its interpretation appropriate?

Yes

Are all the source data underlying the results available to ensure full reproducibility?

Yes

Is the study design appropriate and is the work technically sound?

Yes

Are the conclusions drawn adequately supported by the results?

Yes

Are sufficient details of methods and analysis provided to allow replication by others?

Yes

Reviewer Expertise:

Ethnobotany, Antimicrobial, Antioxidants

I confirm that I have read this submission and believe that I have an appropriate level of expertise to confirm that it is of an acceptable scientific standard.

# Response

**Published:** 2022-08-13

**Authors:** Kevin Githua

**Affiliations:** University of Nairobi, Kenya

Thank you for taking the time to provide these brilliant observations. We have responded to your critique in the order in which you provided the comments.

 1. We have not been able to get any literature that can form a basis for us to hypothesize on the potential causes of weight loss in the experimental animals. The best we have been able to do is captured in the manuscript where we postulated that the decrease in body weight could have something to do with leptin levels (See Alsalahi et al., 2012. Toxicological features of Catha edulis (Khat) on livers and kidneys of male and female Sprague-Dawley rats: A subchronic study. *Evid. Based Complement. Alternat. Med.* 2012; 2012:1–11. 23259000 10.1155/2012/829401).

 Whether the active ingredients in Khat could be the cause of the weight loss is an important question that warrants further inquiry. However, it is beyond the scope of the current study and we would not wish to over-hypothesize this in our discussion.

 2. With regard to the observed damage to the kidney, we have not come across any published material indicating that Cathines/amphetamines or any other components in Khat cause the injuries illustrated in this study. This could be an interesting area for future study.

 3. When you say indication, do you mean adverse effects? If so, we do not believe that our findings contradict what has been observed in humans. We would like to refer you to the study by Al-Meshal et al 1985 (Al-Meshal IA, Ageel AM, Parmar NS, et al.: Catha edulis (khat): use, abuse and current status of scientific knowledge. *Fitoterapia.* 1985) which we used in our discussion where it was established that *Catha edulis* produced changes in the weight, fat mass, appetite, lipid biochemistry, and hormonal levels in humans.

# Reviewer report: Reviewer response for version 1

**DOI:** 10.5256/f1000research.120721.r127808

**Published:** 2022-03-23

**Authors:** Kishwar Ali

**Affiliations:** 1 College of the North Atlantic-Qatar, Doha, Qatar

The article “Studies on the ethnopharmacology, antimicrobial activity, and toxicity of Catha *edulis* (Vahl.) Endl., in *Sprague Dawley* rats” provides a very good insight into ethnobotanical practices and their reality to modern medicines. I believe that it contributes to science by linking sociology, ethnobotany, and pharmacology.

 The study aims to document the ethnopharmacological and antimicrobial properties, and test the toxicity of *C. edulis* leaves collected from the Mbeere South community in Kenya. A good mix of questionnaire and laboratory methods were used which created some beautiful quantitative data, which was interpreted very nicely in graphical and tabulated form.

 The results of the study are really interesting and could be used in the country’s future studies of C. *edulis *and its possible use in the Pharmaceutical industry. All that said above, the manuscript still needs some minor language editing and improvements.

 Some of the issues are highlighted below:

1. In the Abstract, Results, it says…all informants were male, married… In Table 1. 8.6% were single.
2. In the Introduction, Catha *edulis* is a small shrub, but it can reach up to 26 m in height. Isn't it too high for a shrub? Correction needed.
3. Data analysis heading… CogStat is an alternative….which could be used. (Was it used? Or is it just a suggestion?)
4. On Page 8, can Table 1 be separated from the text below it, it appears like this is the caption of the table.
5. Tables placement is a bit confusing, Tables 5 and 6 are mentioned long before they appear in the text.
6. Page 9, can the text” No statically significant….. or high doses of Catha *edulis*) be made more concise? There is a lot of repetitions.
7. Some of the in-text citations are with dates, some are not. Can it be consistent across?

Is the work clearly and accurately presented and does it cite the current literature?

Yes

If applicable, is the statistical analysis and its interpretation appropriate?

Yes

Are all the source data underlying the results available to ensure full reproducibility?

Yes

Is the study design appropriate and is the work technically sound?

Yes

Are the conclusions drawn adequately supported by the results?

Yes

Are sufficient details of methods and analysis provided to allow replication by others?

Yes

Reviewer Expertise:

Phytochemistry, Ethnobotany, antimicrobial activities.

I confirm that I have read this submission and believe that I have an appropriate level of expertise to confirm that it is of an acceptable scientific standard.

# Response

**Published:** 2022-08-13

**Authors:** Kevin Githua

**Affiliations:** University of Nairobi, Kenya

Dear Ali,

 Thank you for taking the time to critique our manuscript and for providing valuable feedback. We have responded in the order in which you critiqued the manuscript i.e. 1-7.

 1. Thank you for this observation. We have since rectified this and it now reads 'All informants were male, most were married'......See the abstract section.

 2. Thank you for this observation. We have since rectified the statement which now reads ' *Catha edulis (Vahl) *is a small shrub with glossy green leaves, a whitish bark, and a small crown...See the opening line of the introduction.

 3. Thank you for your observations. At the time of our initial submission, we were requested by the editorial team at F1000Research to provide an alternative open source software that could potentially be used to analyze this data. Cogstat is one such software. However, please note that we carried out the data analysis using Genstat statistical software and have thus deleted the statement on Cogstat.

 4. Thank you. Please note that there is no text below table 1. Kindly re-check again and confirm.

 5. Thank you. This is not accurate, We have presented Tables 5 and 6 and then gone on to describe the contents of these tables in the text. Kindly re-check and confirm.

 6. Thank you for this observation. We have made the statements more concise by comparing the treatment groups with the control group only. Please see the text below in tables 5 and 6.

 7. Thank you for your observation. For uniformity, we have deleted the dates which appeared in some of the references e.g. reference 4, 7, 10, 16, 23, 24, 28, and 34.
